# Iliopsoas Abscess Due to Nephrolithiasis and Pyelonephritis

**DOI:** 10.5811/cpcem.2018.4.38206

**Published:** 2018-05-18

**Authors:** Daniel Frank, Brian Neal, Andrew Jacobs

**Affiliations:** * Southside Hospital, Department of Emergency Medicine, Bay Shore, New York; † Southside Hospital, Department of Family Medicine, Bay Shore, New York

## CASE PRESENTIATION

A 52-year-old-male presented to our emergency department with gradually worsening left lower back pain radiating to his left leg for six weeks. He had sustained a previous spinal cord injury from a motor vehicle accident with thoracolumbar fusion, and he self-catheterizes for urine due to a neurogenic bladder. He originally attributed his pain to his chronic ailments, but worsening pain and subjective fevers prompted his visit. He presented febrile to 38.6° Celsius, tachycardic to 130 beats per minute, and normotensive. Physical exam was significant for a firm, tender nodular mass to his left flank. Laboratory analysis revealed white blood cell count (WBC) 23.8 × 10(3)/uL, lactate 0.8 mmol/L, and urinalysis demonstrated bacteria too numerous to count and 50 WBC per high-power field.

**Image 1:**
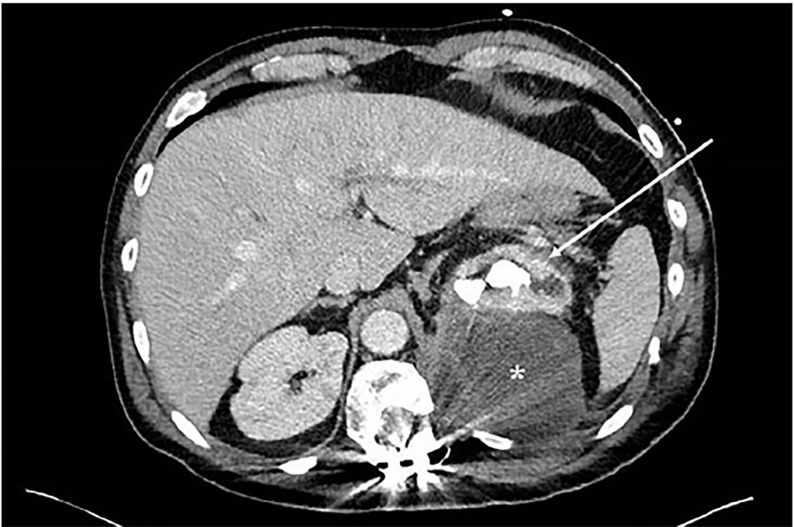
Computed tomography, axial image, demonstrating large fluid collection (*) centered over the left psoas. The left kidney is displaced and demonstrates a staghorn calculus with cystic changes contiguous with the fluid collection (arrow). There is artifact from spinal fusion hardware.

**Image 2:**
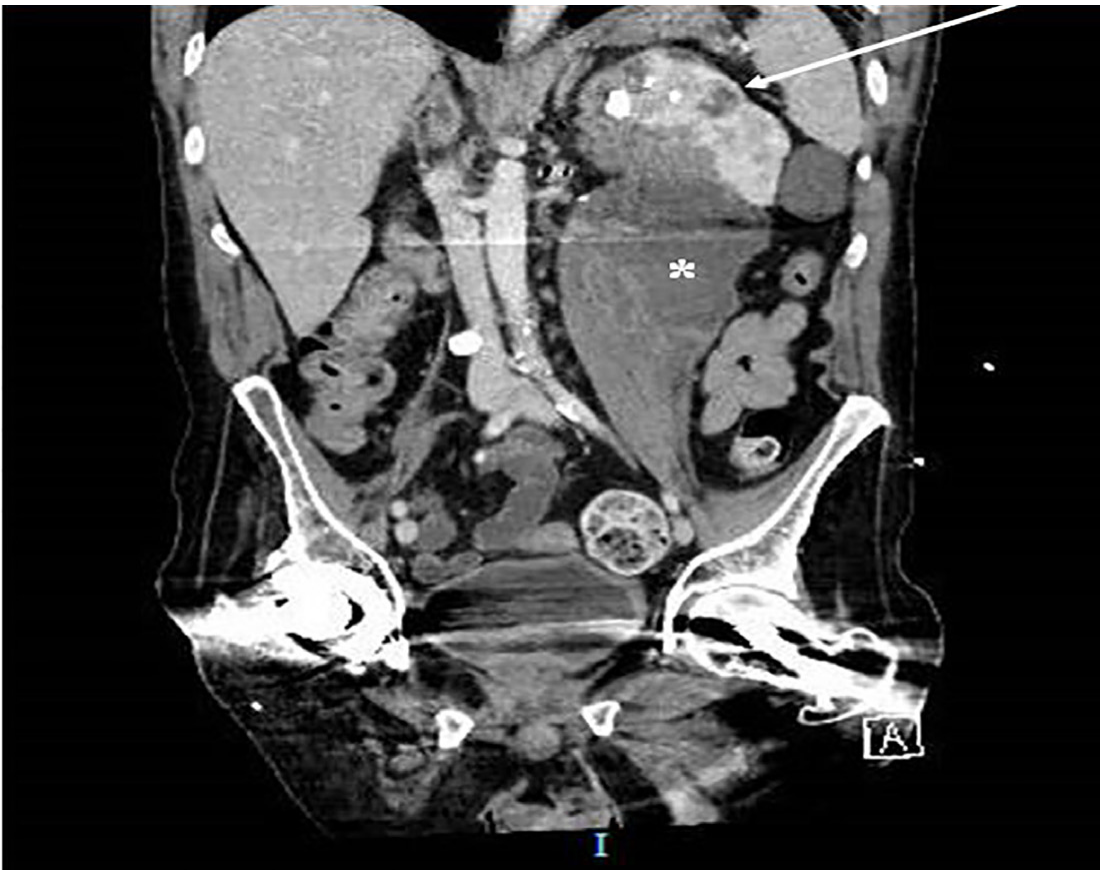
Computed tomography, coronal image, demonstrating large fluid collection (*) centered over the left psoas. The left kidney is displaced and demonstrates a staghorn calculus with cystic changes contiguous with the fluid collection (arrow).

Computed tomography of the abdomen and pelvis revealed a large fluid collection measuring 7.5 × 8.7 × 15 centimeters centered in the left psoas muscle that abuts and displaces the left kidney. The kidney demonstrates a staghorn calculus with surrounding cystic changes that is contiguous with the psoas collection (Images 1 and 2).

Our patient received broad spectrum antibiotics and underwent percutaneous drainage, which is often considered first-line treatment.^1^,^2^ Blood and body-fluid cultures grew beta-hemolytic Streptococcus. He was ultimately discharged with a pigtail catheter left in place and treated for six weeks with intravenous ceftriaxone.

## DISCUSSION

Iliopsoas abscess is a rare condition often presenting with varied and non-specific symptoms. They are often characterized as either primary or secondary. Primary iliopsoas abscess occurs from hematogenous spread, while secondary iliopsoas results from direct extension of a nearby infectious process.^1^ Most secondary cases result from gastrointestinal causes, such as inflammatory bowel disease, diverticulitis, or appendicitis. Cases resulting from genitourinary disease are exceedingly rare, accounting for only 3% of cases in one series.^2^ Our patient was at high risk for genitourinary infections from self-catheterizing. We also discovered a large staghorn calculus that he was previously unaware of. Given that cystic changes in the kidney were contiguous with the collection, the imaging strongly suggests that a chronically infected renal calculus led to formation of the abscess.

Regardless of the cause of a psoas abscess, whether it is primary or secondary, most cases do not require open surgical drainage. In one retrospective review, 97% of patients were treated successfully with percutaneous drainage or antibiotics alone. The authors concluded that the initial management of psoas abscesses should be nonsurgical.^3^

Documented patient informed consent and/or Institutional Review Board approval has been obtained and filed for publication of this case report.

**CPC-EM Capsule**

What do we already know about this clinical entity?

Iliopsoas abscess is a rare clinical entity with various causes and non-specific symptoms. Mainstays of treatment are prompt antibiotics and procedural drainage.

What is the major impact of the image(s)?

Rarely has iliopsoas abscess been attributed to a genitourinary source, nor has such a case been described in the emergency medicine literature.

How might this improve emergency medicine practice?

This case adds to our understanding of the full scope of potential complications related to pyelonephritis and nephrolithiasis, two clinical entities frequently encountered.
